# Free fatty acids induce ER stress and block antiviral activity of interferon alpha against hepatitis C virus in cell culture

**DOI:** 10.1186/1743-422X-9-143

**Published:** 2012-08-03

**Authors:** Feyza Gunduz, Fatma M Aboulnasr, Partha K Chandra, Sidhartha Hazari, Bret Poat, Darren P Baker, Luis A Balart, Srikanta Dash

**Affiliations:** 1 Department of Medicine, Gastroenterology and Hepatology, Tulane University Health Sciences Center, 1430 Tulane Avenue, New Orleans, LA, 70112, USA; 2 Department of Pathology and Laboratory Medicine, Tulane University Health Sciences Center, 1430 Tulane Avenue, New Orleans, LA, 70112, USA; 3 Biogen Idec Inc., Cambridge, MA, 02142, USA

**Keywords:** Hepatitis C virus, Free fatty acids, Hepatic steatosis, IFN alpha, Endoplasmic reticulum stress, Jak-Stat signaling

## Abstract

**Background:**

Hepatic steatosis is recognized as a major risk factor for liver disease progression and impaired response to interferon based therapy in chronic hepatitis C (CHC) patients. The mechanism of response to interferon-alpha (IFN-α) therapy under the condition of hepatic steatosis is unexplored. We investigated the effect of hepatocellular steatosis on hepatitis C virus (HCV) replication and IFN-α antiviral response in a cell culture model.

**Methods:**

Sub-genomic replicon (S3-GFP) and HCV infected Huh-7.5 cells were cultured with a mixture of saturated (palmitate) and unsaturated (oleate) long-chain free fatty acids (FFA). Intracytoplasmic fat accumulation in these cells was visualized by Nile red staining and electron microscopy then quantified by microfluorometry. The effect of FFA treatment on HCV replication and IFN-α antiviral response was measured by flow cytometric analysis, Renilla luciferase activity, and real-time RT-PCR.

**Results:**

FFA treatment induced dose dependent hepatocellular steatosis and lipid droplet accumulation in the HCV replicon cells was confirmed by Nile red staining, microfluorometry, and by electron microscopy. Intracellular fat accumulation supports replication more in the persistently HCV infected culture than in the sub-genomic replicon (S3-GFP) cell line. FFA treatment also partially blocked IFN-α response and viral clearance by reducing the phosphorylation of Stat1 and Stat2 dependent IFN-β promoter activation. We show that FFA treatment induces endoplasmic reticulum (ER) stress response and down regulates the IFNAR1 chain of the type I IFN receptor leading to defective Jak-Stat signaling and impaired antiviral response.

**Conclusion:**

These results suggest that intracellular fat accumulation in HCV cell culture induces ER stress, defective Jak-Stat signaling, and attenuates the antiviral response, thus providing an explanation to the clinical observation regarding how hepatocellular steatosis influences IFN-α response in CHC.

## Introduction

Hepatitis C virus (HCV) infection affects an estimated 180 million people worldwide and is one of the major causes of chronic liver diseases, liver cirrhosis, and hepatocellular carcinoma [1-3]. The standard of care for chronic HCV genotype 1 infection includes a combination of interferon-alpha (IFN-α) , ribavirin and one of the protease inhibitors (either telaprevir or boceprevir). This triple combination therapy has greatly improved the sustained antiviral responses among chronic HCV patients. However, a large percentage of chronic HCV patients are still unable to clear the infection with this regimen. The predictors of sustained virological response (SVR) to interferon based combination therapy have been linked to host genetic factors IL-28B genotypes, viral load, viral genotypes, body weight, stage of liver diseases, obesity, type-2 diabetes mellitus (DM), fibrosis stage and co-infection with human immunodeficiency virus [4-9]. Our understanding of the mechanisms of IFN-α and ribavirin action has significantly increased due to the availability of the HCV cell culture system. We and other groups have shown that IFN-α efficiently inhibits replication of HCV in the cell culture model [10-14]. Therefore, the HCV cell culture model provides an excellent *in vitro* model system to assess the contribution of a number of host related factors in the mechanisms of IFN-α resistance.

A number of clinical studies have reported that overweight or obese HCV-infected individuals or those with steatosis of the liver are at a higher risk for IFN-α non-responsiveness [15-17]. The prevalence of hepatic steatosis in chronic hepatitis C patients has been reported to vary between 50-80%, and is associated with excessive alcohol drinking, increased body weight, DM and other metabolic diseases [18]. The increased lipogenesis and the free fatty acid (FFA) overflow to hepatocytes have been proposed to be the major cause for hepatic steatosis [19]. Chronic HCV infection also leads to abnormalities of lipid metabolism and insulin resistance, factors that also increase the risk of type-2 DM [16]. There are data supporting the fact that patients with high body mass index have a lower chance of SVR [17]. The molecular mechanisms explaining how the hepatic steatosis and related metabolic liver diseases reduce the SVR of IFN-α are unknown.

Palmitic and oleic acids are the most abundant FFAs in liver triglycerides in patients with nonalcoholic fatty liver disease [20]. This study was carried out to examine the effect of co-culturing the mixture of these two FFAs on HCV replication and IFN-α antiviral response using stable sub-genomic replicon and full-length HCV infected cell cultures. We show that FFA treatment of HCV cell culture induces hepatocellular steatosis and lipid accumulation in a dose dependent manner. Intracellular fat accumulation in HCV cell culture increased the viral replication and partially blocked the antiviral response of IFN-α. We present experimental evidence indicating that intracellular lipid accumulation induces ER stress response and down regulates the IFNAR1 chain of the type I interferon receptor, leading to the creation of defective Jak-Stat signaling and impaired antiviral response of IFN-α against HCV.

## Materials and methods

### HCV cell culture and chemicals

The stable S3-GFP replicon cell line (HCV2a) was maintained in Dulbecco’s Modified Eagle’s Medium (DMEM) supplemented with 2 mM L-glutamine, sodium pyruvate, nonessential amino acids, 100 U/mL penicillin, 100 mg/mL streptomycin, and 10% fetal bovine serum supplemented with G-418 (1 μg/mL) [21]. Nile red, sodium oleate, sodium palmitate, and fatty acid free bovine serum albumin (BSA) were obtained from Sigma Chemical Co., Saint Louis, MO. Recombinant human IFN-α 2b (Intron A) was purchased from Schering Plough, Kenilworth, NJ. The Huh-7.5 cell line was obtained from the laboratory of Charlie Rice (The Rockefeller University, New York) and maintained in DMEM with 10% FBS.

### Western blot

Protein lysates from S3-GFP replicon cells were prepared after treatment with FFAs. Equal amounts of protein were resolved on SDS-PAGE gels [21]. The antibodies to Stat1, Stat2, p-Stat1 (Y701), p-Stat2 (Tyr690), p-Jak1 (Tyr1022/1023), p-Tyk2 (Tyr1054/1055), total eIF2α, p-eIF2α (Ser51), beta-actin, IRE1-alpha, PKR, PERK, BIP, SOCS-3, anti-mouse IgG, and anti-rabbit IgG HRP-linked antibody were purchased from Cell Signaling, Beverly, MA. Antibodies to interferon alpha-receptor 2 (IFNAR2) and p-IFNAR1 were purchased from Santa Cruz Biotechnologies, Santa Cruz, CA. The antibody to p-PKR (pT446) was obtained from Epitomics, Burlingame, CA. A mouse monoclonal antibody to IFNAR1 (GB8) was kindly provided by Biogen Idec Inc., Cambridge, MA, USA.

### Fatty acid treatment

We used a formulation of FFA mixture at a 2:1 ratio of oleate to palmitate that mimics benign chronic steatosis with low toxicity described by other investigators [22-25]. Briefly, 100 mM palmitate (Sigma catalog No. P-0500) and 100 mM Oleate (Sigma Catalog No. 0–7501) stocks were prepared in 0.1 M NaOH at 70°C and filter sterilized. Five percent (w/v) FFA-free BSA solution was prepared in double distilled water and filter sterilized. A 5 mM stock solution for each fatty acid was prepared in 5% BSA solution in distilled water at 60°C then the mixture was cooled to room temperature. These two FFAs were first mixed together in growth medium in a sterile environment under laminar flow so that the final concentration of FFA was 1 mM and BSA was 1%. S3-GFP cells were cultured in 10-cm dishes to 80-85% confluence and co-cultured with different concentrations of FFA for 24 hours to induce steatosis. At indicated time points, the cells were washed in PBS and cultured in regular growth medium containing 10% FBS.

### Nile red staining and microfluorometry

Intracellular fat accumulation in the HCV cell culture was determined by Nile red staining as described [22]. Briefly, hepatocyte monolayers were washed twice with phosphate-buffered saline (PBS) and incubated for 15 minutes with Nile red solution at a concentration of 1 mg/mL in PBS at 37°C. After this treatment, the cell monolayer was washed with PBS and counterstained with a nuclear staining Hoechst dye (H33342, Calbiochem, Darmstadt, Germany) at a concentration of 10 μg/mL prepared in PBS. Cells were then examined under a fluorescence microscope as described previously [21]. The intracellular fat content was quantitated using a microfluorometer (excitation 488 nm and emission 550 nm).

### Electron microscopy

Intracellular fat accumulation in the FFA treated cells was also confirmed by electron microscopy using a standard protocol [26].

### MTT assay

The effect of FFA treatment on the cell proliferation was measured using a MTT colorimetric assay [27]. The assay uses a tetrazolium compound (3-[4, 5-dimethylthiazol-2-yl]-2, 5-diphenyl tetrazolium bromide) (M5655, Sigma-Aldrich, St Louis, MO), which is reduced intracellularly to formazan by a mitochondrial dehydrogenase enzyme. The percentage of cell viability was determined by comparison with untreated controls.

### Flow cytometry

The effect of FFA treatment on HCV replication and IFNAR1 expression was measured by flow analysis as described previously [21]. Briefly, S3-GFP replicon cells with or without FFA treatment were collected 24 h post- treatment. Intracellular GFP expression was quantified using a flow cytometer (BD LSR II, BD Biosciences). For the analysis of IFNAR1 expression, Huh-7 cells were treated similarly and processed using an IFNAR1 antibody (Epitomics, Burlingame, CA), as per the supplier’s protocol, with the secondary antibody (Alexa Fluor 488-labeled) procured from Invitrogen, CA. Histograms were generated using WinMDI version 2.8 software (Scripps.edu).

### Real-time RT-PCR

Total RNA was isolated from the cells using the GITC method and real-time RT-PCR was carried out as described [28].

### Infectious cell culture model for HCV with a luciferase reporter

Full-length chimeric virus encoding Renilla luciferase (pJFH-ΔV3-Rluc) was kindly provided by Curt H Hagedorn, University of Utah School of Medicine, Salt Lake City [29]. The pJFH-ΔV3-Rluc plasmid was linearized with *Xba I*, extracted with phenol /chloroform and precipitated with ethanol. HCV-RNA transcripts were prepared *in vitro* by using T7 RNA polymerase. 1 × 10^7^ Huh-7.5 cells were electroporated with 20 μg of HCV RNA. Transfected cells were cultured in complete DMEM. Infectivity assay was carried out using the supernatants 96 hr post-transfection. Briefly, Huh-7.5 cells were infected with JFH-ΔV3-Rluc virus (MOI 0.1) overnight. On the following day, the infected culture was washed with PBS and then incubated with 10 mL of DMEM with 10% FBS. Infected Huh-7.5 cells were cultured long-term by splitting at a 1:10 ratio at five-day intervals. Replication of HCV in the infected cell culture at each interval was confirmed by measuring the *Renilla* luciferase activity or the HCV RNA level by RT-qPCR. Renilla luciferase activity was measured in 5μL cell lysate using a luciferase assay kit (Promega) and total protein was measured using the Bradford assay.

### Luciferase assay

The effect of FFA treatment on Jak-Stat signaling and IFN-β (pISRE-Firefly luciferase) promoter activity was examined using a published protocol (21). The ATF6-Luc activity was measured by co-transfecting 0.5 μg of p5XATF6GL3 (Addgene, Cambridge, MA) DNA along with pRL-TK plasmid (0.5 μg) in S3-GFP cells 24 h prior to FFA treatment. Cell lysates were prepared 24 h post FFA treatment and the Firefly luciferase values were normalized to the Renilla luciferase values.

### Immunohistochemical staining for HCV-core protein

Infected Huh-7.5 cells were mounted onto a glass slide via the cytospin method. The cells were washed twice with 10 mM PBS pH 7.4 (Sigma-Aldrich, St Louis, MO) for 5 minutes. The cells were fixed in chilled acetone for 15 minutes and then permeabilized by treatment with Reveal Decloaker RTU (Biocare Medical, RV 100) for 25 minutes at boiling point. Slides were then cooled down at room temperature for 25 minutes. Blocking was performed utilizing Background Sniper (Biocare Medical, BS966) for 10 minute at room temperature. The cells were incubated with monoclonal anti-core antibody (Thermo Scientific, Pierce hepatitis C virus core antigen specific mouse monoclonal antibody, Ma1-080) at 1:200 diluted with Da Vinci Green Diluent (Biocare Medical, PD900) for 1 hour at room temperature. Following the primary antibody incubation, the cells were washed 3 times in Tris Buffered Saline (pH 8.0), and incubated with MACH 4 mouse probe (Biocare Medical, UP534) for 10 minutes. After mouse probe treated, the cells were incubated with MACH4 HRP Polymer (Biocare Medical, MRH534) for 30 minutes, and the cells were washed with TBS 3 times. Next, the cells were treated with diaminobenzidine (DAB) chromogen (Dako Cytomation, Carpinteria, CA) for 5 minutes. The slides were counterstained with hematoxylin for 30 seconds and Tacha’s bluing Solution (Biocare Medical, HTBLU) for 30 seconds, dehydrated, mounted and observed by light microscopy.

### Statistical analysis

Microfluorometry assay results, ISRE, ATF6 (activating transcription factor 6), and HCV (pJFH-ΔV3-Rluc) luciferase activity, real time RT-PCR and FACS analysis were compared for significance using the Student’s *t* test. *P* values less than 0.05 were considered significant.

## Results

### FFA treatment induces cellular steatosis in HCV cell culture

**Figure 1:**
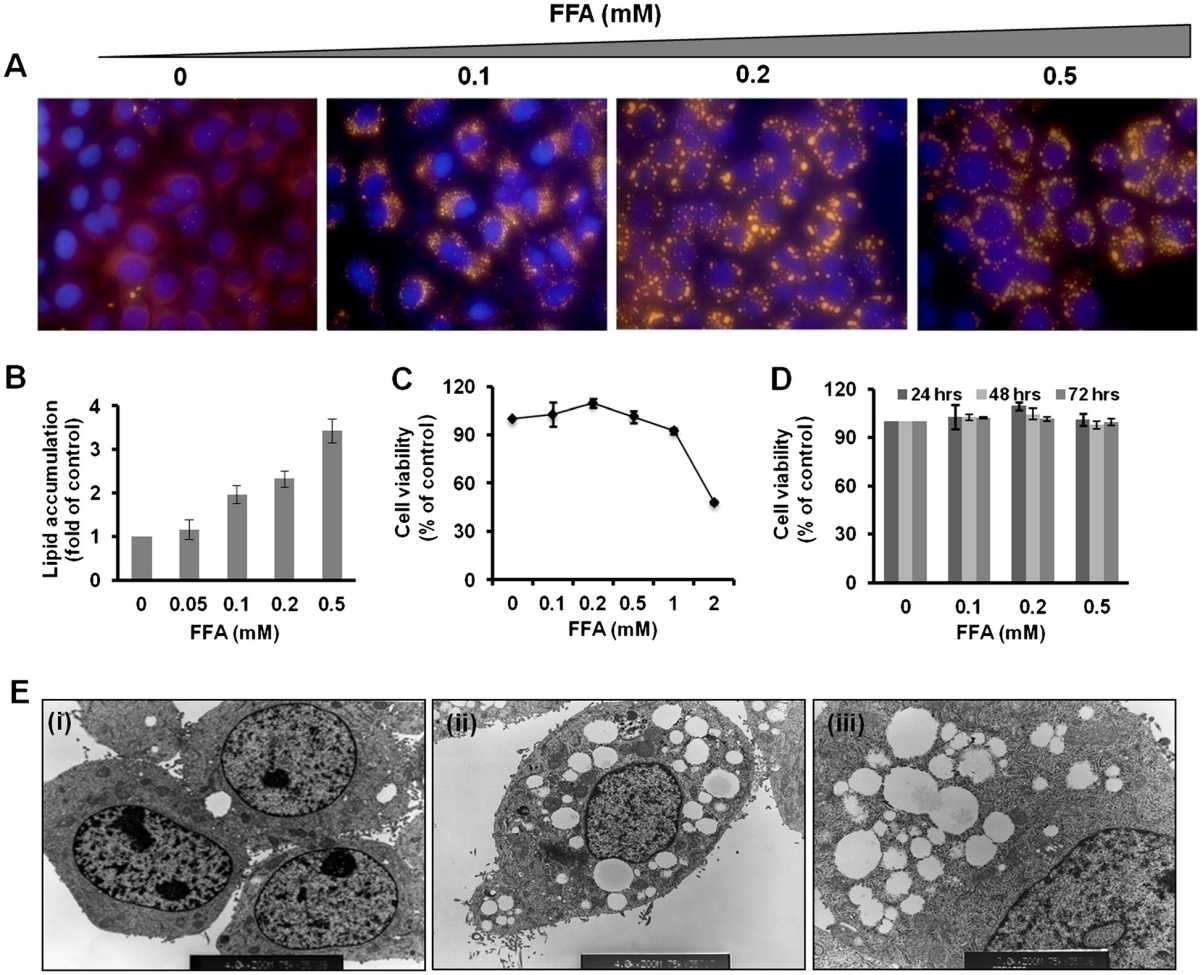
** FFA treatment induces hepatocellular steatosis in HCV replicon cells.** S3-GFP cells in culture were treated with increasing concentrations of Oleate/Palmitate (2: 1 ratio) and hepatocellular steatosis due to an intracellular fat accumulation was characterized by a number of methods. (**A**) Fluorescence microscopy showing concentration dependent hepatocellular steatosis in S3-GFP cells 24 h post treatment by Nile Red staining (yellow) and nuclear staining (blue). The images were taken at 40× magnifications. (**B**) Microfluorometer analysis of intracellular fat accumulation in S3-GFP cells 24 h after FFA treatment. The values are expressed as fold change compared to untreated cells, *P <0.001, Student *t* test. (**C**) MTT assay showing the effect of intracellular fat accumulation (dose-dependent) on cellular cytotoxicity of S3-GFP cells in culture. Cell viability was expressed as % of untreated. (**D**) MTT assay showing cell viability over time (h) for concentrations of FFA up to 0.5 mM. (**E**) Electron micrograph of FFA-treated (0.5 mM) S3-GFP cells showing intracellular lipid droplet accumulation. (i) Untreated cells, (ii) FFA-treated cells, 4000×magnification, and (iii) 7000× magnification showing fat droplets in the endoplasmic reticulum (ER) (black arrow).

Oleate (unsaturated) and palmitate (saturated) fatty acids were mixed at a ratio of 2:1 and directly added to the cell culture medium supplemented with 1% BSA to induce steatosis in S3-GFP cells in culture. The concentration of saturated fatty acids to nonsaturated fatty acids used in our study is comparable to the pathological range of human non-alcoholic fatty liver disease. First, S3-GFP cells were cultured with increasing concentrations of FFA and hepatocellular steatosis was confirmed by fluorescence microscopy after Nile red staining. Representative pictures show that FFA treatment resulted in dose-dependent intracellular lipid accumulation in the HCV replicon cells that can be visualized by fluorescence microscopy (Figure 1A). S3-GFP cells treated with an equimolar concentration of BSA carrier protein were used as a control. Results of microscopic findings in lipid droplet accumulation in the cytoplasm of HCV cell cultures were quantified by microfluorometry (Figure 1B). The concentration-dependent toxic effect of FFA treatment in the HCV cell culture was determined by using the MTT assay (Figure 1C) indicating that increasing concentrations of FFA were toxic to the cells. MTT assay results showed that the FFA mixture caused cellular toxicity at and above 1 mM. The long-term stability and toxicity of intracellular lipid droplet accumulation in the FFA treated HCV cell culture was also examined in a kinetic study (Figure 1D) suggesting that FFA up to 0.1 to 0.5 mM can induce a fairly high level of hepatocellular steatosis in 100% of the cells in culture without causing apparent toxicity. Electron microscopic studies confirmed that S3-GFP cells cultured with FFA developed intracytoplasmic accumulation of lipid droplets in the vicinity of the ER (Figure 1E). Based on these results, hepatocellular steatosis in HCV cell culture was carried out using the highly viable concentrations of FFA (10–100 μM) to determine its impact on virus replication and IFN-α antiviral response.

### Intracellular fat accumulation increases HCV RNA replication

**Figure 2:**
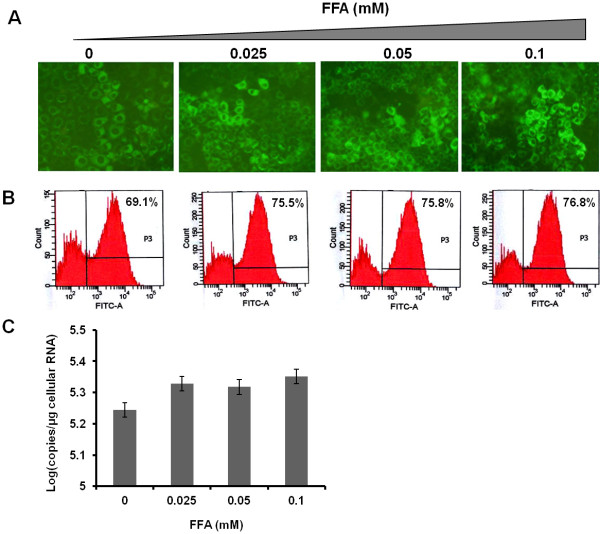
** Effect of hepatocellular steatosis on HCV RNA replication in S3-GFP replicon cell line.** S3-GFP replicon cells were cultured with different concentrations of FFA in 1% BSA for 5 days. Untreated cells received only 1% BSA. (**A**) The HCV-GFP expression was measured by using fluorescence microscopy. (**B**). Quantification of GFP expression of S3-GFP in replicon cells by flow analysis. The number indicates the mean fluorescence of GFP-positive cells. (**C**). Intracellular HCV RNA levels in S3-GFP replicon cells were measured by real time RT-PCR.

**Figure 3:**
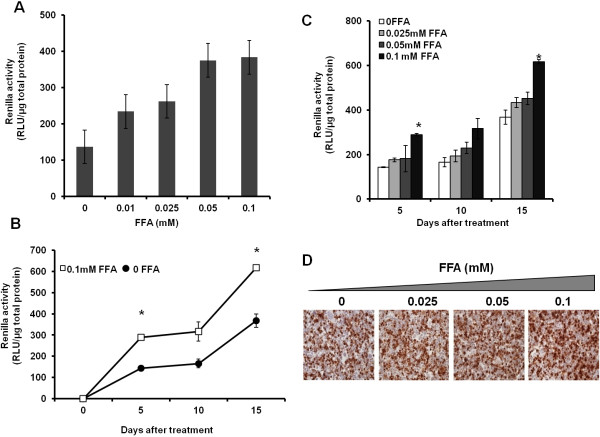
** Effect of hepatocellular steatosis on HCV RNA replication in the infected Huh 7.5 cells.** Huh-7.5 cells were infected overnight with HCV chimeric virus encoding Renilla luciferase (pJFH-ΔV3-Rluc). Infected Huh-7.5 cells were cultured long-term in growth media by splitting at a 1:10 ratio at five days intervals. (**A**). HCV-Renilla luciferase activity of infected Huh-7.5 cells cultured in the presence FFA for 72 hrs. The values in the y-axis represent the normalized Renilla luciferase values per one μg of total protein. The X-axis shows the different concentrations of FFA. (**B**). Effect of long-term culture FFA on HCV replication. HCV infected cells were cultured in the presence of 100 μM of FFA for 5, 10 and 15 days, the level of HCV RNA was measured by luciferase assay. The statistical significance was determined by paired T-test, the stars indicate a p value <0.05. (**C**). HCV infected cells were cultured in the presence of 100 μM concentrations of FFA for 5, 10 and 15 days. HCV replication was measured by luciferase assay. The statistical significance was determined by paired T-test, the stars indicate a p value <0.05. (**D**) Immunohistochemical staining for HCV core antigen in the infected Huh 7.5 cells in the presence of different concentrations of FAA after 15 days.

To determine whether intracellular fat accumulation plays a role in modulating HCV RNA replication, we cultured S3-GFP replicon cells with different concentrations of FFA (25-100 μM). The expression of HCV GFP fusion protein was monitored using fluorescence microscopy (Figure 2A), and then quantified by flow cytometric analysis (Figure 2B). The mean fluorescence of GFP-positive cells following FFA treatment for 5-days was increased from 69.1% to 76.8% as compared to cells treated with BSA (Figure 2B). The increase in HCV RNA levels in the S3-GFP cells after treatment with increasing concentration of FFA after 5-days was measured by real time RT-PCR (Figure 2C). The replicon based HCV cell culture model lacks the structural protein and this culture does not produce an infectious virus, therefore the effect of FFA treatment on HCV replication was examined using a persistently infected HCV cell culture system. The replication of HCV in the infected Huh-7.5 cells after FFA treatment was measured using a Renilla luciferase reporter. Cells were infected with a cell culture derived virus (MOI of 0.1) by overnight incubation then maintained in a long-term culture by splitting at 1:10 ratio. The effect of the long-term and short-term culture of FFA on HCV replication was measured. Initially, we determined the dose dependent effect of FFA treatment on HCV replication in the infected culture short term over 72 hours. The results indicate intracellular fat accumulation in the infected cell culture which resulted in a dose dependent increase in HCV replication measured by Renilla luciferase activity (Figure 3A). A second set of experiments was performed to determine the effect of long-term co-culture of FFA on HCV replication in the infected cell culture. For this purpose, persistently infected cells were cultured with the FFA for 5, 10 and 15 days then HCV replication in the culture with or without FFA treatment was measured by Renilla luciferase activity. Results of this experiment show a statistically significant increase in the HCV replication with a concentration of 100 μM of FFA (Figure 3B &C). The effect of FFA treatment on HCV replication in the infected Huh-7.5 cells was also confirmed by immunostaining for core protein. Results shown in Figure 3D indicate that HCV core immunostaining of persistently infected Huh-7.5 cells that had been cultured with the FFA for 15 days show intense core staining as compared to those without FFA treatment. Taken together our results indicate that long-term culture of FFA with infected cells led to an increased HCV replication.

### Intracellular lipid accumulation blocks IFN-α antiviral response against HCV

**Figure 4:**
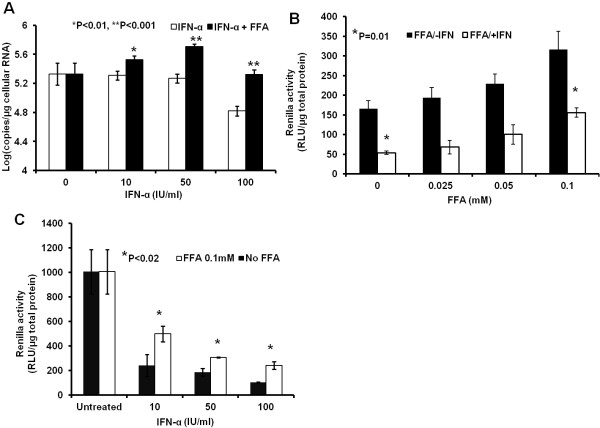
** FFA treatment blocks the antiviral response of IFN-α against HCV.** S3-GFP and infected Huh-7.5 cells were cultured with FFA for 5 days and then treated with IFN-α for 72 hours. (**A**) The antiviral effect of IFN-α with and without FFA treatment measured by real-time RT-PCR in S3-GFP replicon cells. *P < 0.01, **P < 0.001 , Student *t* test. (**B**) Renilla luciferse activity showing the antiviral effect of IFN-α in the infected Huh-7.5 cells co-cultured with increasing concentration of FFA. The values are normalized with total protein. (**C**) Luciferase activity showing the dose dependent antiviral effect of IFN-α blocked by FFA treatment (100 μM) in the infected Huh-7.5 cells. The values in the y-axis represent the values (RLU) normalized per μg of protein lysates**.** The values in the x-axis represent the different concentrations of IFN-α (IU/ml).

We tested whether intracellular lipid droplet accumulation affected IFN-α responsiveness to HCV replication using both a replicon and an infected cell culture model. S3-GFP replicon cell line was cultured in growth medium with or without FFA for 5 days, after which they were treated with IFN-α for an additional 72 h. S3-GFP cells were also co-cultured with FFA while treated with IFN-α. The titer of HCV RNA in the replicon culture was quantified using a real-time RT-PCR assay indicating that FFA treatment partially blocks the IFN-α antiviral effect against HCV in a concentration dependent manner (Figure 4A). The effect of FFA treatment on the IFN-α antiviral response was confirmed using a persistently infected HCV cell culture model. Infected Huh-7.5 cells were co-cultured with different concentrations of FFA (0–100 μM) then for 5-days after that, the cultures were treated with IFN-α for 72 h. Replication of HCV in the infected cell culture model was examined by Renilla luciferase assay. Results shown in Figure 4B indicate that FFA treatment blocked IFN-antiviral response in a dose dependent manner. Infected cells treated with FFA show a dose dependent increase in Renila luciferase activity. The concentration dependent IFN-α antiviral effect against HCV in the infected cell culture was examined in the presence and absence of 100 μM FFA treatment (Figure 4C). In summary, our results support that FFA treatment blocked antiviral action of IFN-α in replicon and infected cell culture. The results are statistically significant.

### FFA induces ER stress to down regulate IFNAR1 and blocks Jak-Stat signaling

**Figure 5:**
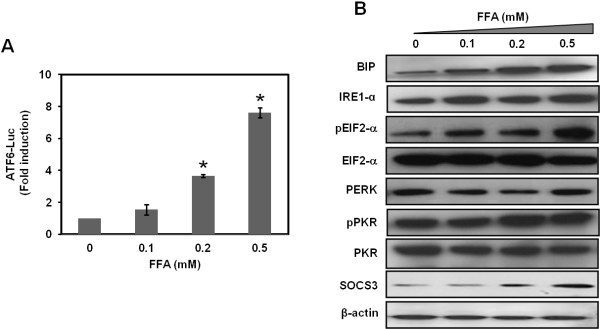
** FFA treatment induces an ER stress response in S3-GFP replicon cells.** (**A**) ATF-6 firefly luciferase activity was measured in S3-GFP cells treated with different concentrations of FFA for 24 h. 24 h post transfection, luciferase activity was measured and values are expressed as fold induction of control. *P < 0.05, Student *t* test. (**B**) Western blot analysis showing that different concentration of FFA induces BIP, IRE1-α, phospho PKR, phospho eIF2α, and SOCS3 in S3-GFP replicons. β-actin levels were used as loading controls.

**Figure 6:**
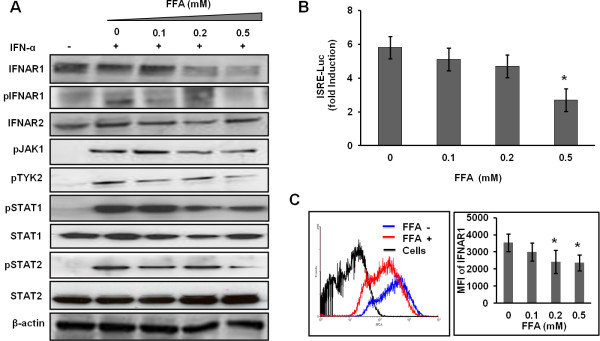
** FFA treatment of S3-GFP replicon cell culture blocks IFN-α induced Jak-Stat signaling.** (**A**) Western blot analysis of Jak-Stat signaling proteins were done in S3-GFP cells cultured with different concentration of FFA for 24 h and then treated with 1000 IU/mL IFN-α for 30 minutes. β-actin levels were used as loading controls. (**B**) FFA treatment blocks IFN-α-induced ISRE-firefly luciferase activity. S3-GFP cells were transfected with 1 μg of pISRE-luciferase plasmid and then cultured with FFA for 24 h. Cells were then treated with 1000 IU/mL IFN-α. 24 h post-transfection, IFN-β promoter activity in the presence and absence of FFA treatment was measured. *P < 0.05 (1 tail), Student *t* test. (**C**) Flow cytometric analysis of IFNAR1 expression in Huh-7 cells treated with or without FFA. The left panel shows a histogram of IFNAR1 expression with FFA (0.5 mM) treatment for 24 h shifted to the left (red line). The right panel shows the mean fluorescence intensity (MFI) of the IFNAR1 signal. The flow cytometry plot is representative of three separate experiments, while the MFI values shown are an average (±SD) of these experiments.

To find an explanation for why S3-GFP replicon cells that were cultured with FFA showed an impaired IFN-α antiviral effect, we examined the ER stress pathway. Recent published reports suggest that FFA treatment induces an ER stress response [30]. Therefore, the activation of three independent ER stress pathways including PERK (RNA-dependent protein kinase-like endoplasmic reticulum eIF2a kinase), IRE1 (inositol requiring enzyme 1), and ATF6 (activating transcription factor 6) were examined [31]. Results shown in Figure 5A indicated that ATF6-firefly luciferase was activated in a dose-dependent manner in FFA-treated S3-GFP cells compared to untreated cells. FFA treatment of S3-GFP cells induced the ER stress related markers BIP, IRE1-α, and p-eIF2α (Figure 5B). The levels of other kinases like PERK and PKR did not alter with FFA treatment. FFA treatment also increased SOCS3 (suppressor of cytokine signaling 3) levels. To explain the potential mechanisms that connect the ER stress response to defective Jak-Stat signaling, we examined the cell surface expression of IFNAR1, which is a known target of ER stress mechanisms. The expression level of IFNAR1 in S3-GFP replicon cells with or without FFA treatment was examined by Western blot analysis and flow cytometry. Results shown in Figure 6, indicate that FFA treatment resulted in reduced expression of IFNAR1 but not of IFNAR2. We then examined the potential effect of ER stress response of FFA on IFN-induced Jak-Stat signaling by measuring phosphorylation of downstream proteins including IFNAR1, Jak1, Tyk2, Stat1, and Stat2 by Western blot analysis (Figure 6A). Tyk2 phosphorylation was affected significantly as this is dependent on IFNAR1 expression, but phosphorylation of pJak1 was unaltered. Intracellular Jak-Stat signaling in FFA-treated cells was also examined using a firefly luciferase reporter plasmid driven by the IFN-β promoter. ISRE-Luc promoter activity was significantly affected by FFA treatment (Figure 6B). The histogram of IFNAR1 cell surface expression showed a change in the peak (shift to left) of FFA-treated cells (Figure 6C) and the mean fluorescence intensity (MFI) for FFA-treated cells decreased at 0.2 and 0.5 mM FFA. These results suggest that FFA treatment blocks IFN-α mediated activation of IFN-β promoter activity in S3-GFP cells and thus impairment of antiviral action. 

## Discussion

The standard of care for chronic HCV genotype 1 infection includes IFN-α plus ribavirin along with one of the protease inhibitors. However, results of clinical studies indicate that the sustained virologic response of this combination therapy is impaired by viral and host related factors. Viral factors play an important role in the treatment response since patients infected with HCV genotype 1 show poor response as compared to genotype 2 and 3 [32,33]. In addition to virus genotype, several host-related factors can also affect the outcome of the antiviral therapy including viral load, presence of cirrhosis, age, race, and metabolic diseases such as obesity and diabetes [34,35]. Obesity is a risk factor resulting in a poor treatment response to both pegylated interferon and pegylated interferon in combination with ribavirin [15-17]. Hepatic steatosis can develop secondary to obesity, DM, alcohol abuse, protein malnutrition, carbohydrate overload, and chronic HCV infection [36]. Hepatic steatosis is also a common histopathological feature of chronic HCV infection that is found in 30-70% of patients [37,38]. There are reports indicating that HCV infection induces the development of hepatocellular steatosis by blocking the release of very low-density lipoprotein (VLDL) particles from the liver to the circulation [38]. It has been reported by a number of investigators that the presence of hepatic steatosis in patients with chronic HCV infection affects liver disease progression, pathogenesis, and treatment response [39-43]. The mechanisms of the impaired response to interferon-based therapy in the condition of hepatic steatosis are not clearly understood.

We took advantage of the HCV cell culture system established in a liver-derived cell line to study the mechanisms of IFN-α antiviral response in the presence or absence of FFAs. Hepatocellular steatosis was induced in HCV replicon cells with a mixture of saturated and non-statured FFAs. Other investigators have used this FFA cocktail to study the pathogenic mechanism of hepatic steatosis in cell culture. Our results support that FFA treatment can induce steatosis in HCV replicon cells in a dose-dependent manner. High dose FFA treatment in HCV cell culture leads to increased cell toxicity and cell death by apoptosis as reported by others [23,30]. We show that the FFA at 10–100 μM range increased HCV replication in the infected cell culture supporting data published previously [44]. Our results suggest that intracellular fat accumulation partially blocks IFN antiviral action and viral clearance in replicon and infected cell culture. Published reports from our laboratory and others indicate that cellular Jak-Stat signaling is critical for the successful antiviral response of IFN-α against HCV [45]. Our results provide evidence supporting that FFA treatment of HCV cell culture induces an ER stress response that blocks cellular Jak-Stat signaling by down regulating IFNAR1. As a result, IFN-α induced Stat1, Stat2 phosphorylation, and IFN-β promoter activity was attenuated. Studies by other laboratories, including ours, have shown that ER stress is correlated well with down regulation of IFNAR1 in cell culture models [45-47]. We propose that future studies should be conducted to determine if attenuating the ER-stress response by pharmacological inhibitors or siRNA knockdown improves the antiviral response of IFN-α in the condition of hepatic steatosis by retained expression of IFNAR1.

**Figure 7:**
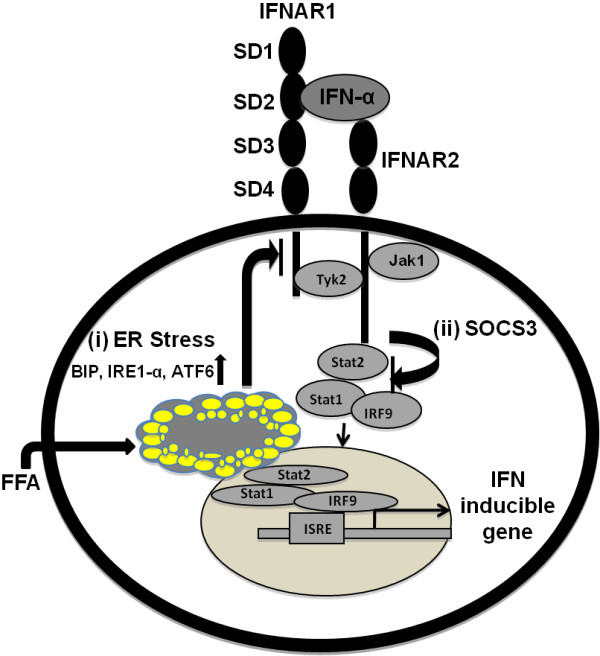
** Schematic representation showing that intracellular lipid droplet accumulation affects Jak-Stat signaling by two possible mechanisms.** (i) Intracellular fat accumulation induces the ER stress related proteins ATF-6, BIP, IRE1-α, and this causes phophorylation of eIF2 α which leads to the down regulation of IFNAR1 expression. The reduced expression of IFNAR1 affects IFN-α binding, Jak-Stat signaling, and downstream antiviral responses. IFN-α binds to its cell surface receptor that activates Jak1 and Tyk2 phosphorylation. The activation of Jak1 and Tyk2 is required for the Stat1 and Stat2 phosphorylation. pStat1 and pStat2 bind to IRF9 to form a complex that translocates to the nucleus to induce IFN-stimulated gene expression. (ii) We show here that FFA treatment induces SOCS3 that also inhibits the phosphorylation of Stat1 and Stat2.

These results using HCV cell culture provide an explanation as to the mechanism by which chronic HCV patients with fatty liver show an impaired response to IFN and ribavirin treatment. In regards to our findings, we propose a model that chronic HCV patients with steatosis have increased lipid droplets in hepatocytes that block interferon-dependent Jak-Stat signaling (Figure 7). Our results are also supported by a number of studies where the role of IFNAR1 expression has been correlated with the response to IFN-α therapy in chronic hepatitis C (48–52). The studies conducted by Taniguchi et al., [48] indicate that high intrahepatic mRNA levels of IFNAR1 among chronic HCV 1b patients before treatment is associated with a favorable response to IFN therapy. Another study by Katsumi et al. [49] reported that the expression rate of IFNAR1 and IFNAR2 were significantly higher in responders than non-responders. Fujiwara et al. [50] have conducted a study where the expression of IFNAR1 receptor and response to interferon therapy was examined in chronic hepatitis C patients. They found the IFNAR2 expression level in the liver is predictive of the response to IFN-α treatment in chronic hepatitis C patients. A study by Meng et al. [51] also examined the expression of IFN-α and β receptor in the liver of patients with chronic hepatitis C who are IFN responders and nonresponders. The authors found that the expression of the interferon receptor was more obvious in the IFN-α treatment responsive group than in the non-responsive group. Welzel et al. [52] have analyzed the relationship between variants in the IFN-α pathway and SVR among participants in the hepatitis C antiviral long-term treatment against the cirrhosis (HALT-C) trial. They found statistical significance in the IFNAR1 expression and that the IFNAR2 expression is associated with a response to antiviral therapy of chronic HCV patients. In addition to this, a number of studies have provided evidence suggesting that other mechanisms may be involved in the impaired response of IFN-α in obese patients. For example, Walch et al. [17] found that increased expression of SOCS3 protein is associated with non-response to IFN-α therapy. These investigators proposed that increased SOCS3 expression also blocks tyrosine phosphorylation of Stat1 in response to IFN-α stimulation. We also found that SOCS3 levels are increased but SOCS1 are not increased (data not shown) in replicon cells treated with FFA. The involvement of SOCS3 is also another possible mechanism for how the intracellular lipid alters Jak-Stat signaling. These in vitro findings suggest that FFA-induced ER stress and SOCS3 levels are the two major targets that play a role in reducing Jak-Stat signaling and impaired antiviral response of IFN-α in FFA-treated cells. 

## Competing interests

The authors declare that they have no competing interests.

## Author contributions

FG and FA performed major biochemical experiments, participated in the design of the study and wrote the initial draft of the manuscript. PKC, SH and BP did some biochemical experiments. DPB provided critical reagents and revised the manuscript. LAB and SD supervised, helped to design the study and wrote the manuscript.
